# Local Causes of Disease

**Published:** 1870-09

**Authors:** 


### Editorial Department.

### Local Causes of Disease.

WE have been observing the prevailing diseases of Buffalo for the season thus far as a test of the generally received hygienic laws which are supposed to govern the health of cities. Sewerage exposed to evaporation during the hot season, street and out-house accumulations, and similar decomposing and offensive sources of atmospheric contamination are now universally believed to be fruitful sources of disease, and as we have had the most favorable opportunities for testing the soundness of this opinion, during the past few months, it would be proving ourselves untrue to hygienic science if no record is made of the observation. The sewerage of nearly one-half of the city has for years passed through an uncovered canal extending for a mile or more in the very heart of the city. For the past season the offensive smell from this canal has been generally perceptible over a considerable portion of the more thickly settled part of the city, while in those parts immediately contiguous it has been almost insufferable; the atmosphere hardly capable of sustaining animal life.

Believing that facts would certainly support our hygienic theories, we naturally looked for the visitation of a plague that should depopulate all that portion of the town and extend its ravages to citizens who resided elsewhere but were doing business near the plague spot. Mankind can live and be healthy in the vilest places, inhaling continually the most offensive and disgusting vapors, or a great number of our people are, have been, or will be sick. We *must* be sick in Buffalo, very sick indeed, or must re-write our principles of hygiene, either of which is attended with serious objections. The question then hinges upon facts and the inquiry is made: Has the effluvia of said canal produced any noticeable and manifest disease? Has it increased the usual death rate, and have the inhabitants of this portion of the city suffered any more from disease than other and apparently more healthy portions of the city?

From personal observation we believe typhoid fever more prevalent than usual, but we look for the official record of diseased, and the observation of others to determine whether the great fountain of all uncleanliness has really been productive of any disease whatever. The general and unusual prevalance of typhoid fever cannot probably be attributed to this cause, since cases are as common elsewhere as in the immediate vicinity. Has it been productive of diarrhœa or dysentery or any other form of disease? What have the profession to say about it?

